# Leaks in the Pipeline: a Failure Analysis of Gram-Negative Antibiotic Development from 2010 to 2020

**DOI:** 10.1128/aac.00054-22

**Published:** 2022-04-26

**Authors:** Neha K. Prasad, Ian B. Seiple, Ryan T. Cirz, Oren S. Rosenberg

**Affiliations:** a Chan Zuckerberg Biohub, San Francisco, California, USA; b Department of Medicine, University of California, San Francisco, San Francisco, California, USA; c Department of Pharmaceutical Chemistry, University of California, San Francisco, San Francisco, California, USA; d Cardiovascular Research Institute, University of California, San Francisco, San Francisco, California, USA; e Revagenix Inc., San Mateo, California, USA; f Department of Biochemistry, University of California, San Francisco, San Francisco, California, USA

**Keywords:** Gram-negative, antibiotics, clinical trials, toxicity

## Abstract

The World Health Organization (WHO) has warned that our current arsenal of antibiotics is not innovative enough to face impending infectious diseases, especially those caused by multidrug-resistant Gram-negative pathogens. Although the current preclinical pipeline is well stocked with novel candidates, the last U.S. Food and Drug Administration (FDA)-approved antibiotic with a novel mechanism of action against Gram-negative bacteria was discovered nearly 60 years ago. Of all the antibiotic candidates that initiated investigational new drug (IND) applications in the 2000s, 17% earned FDA approval within 12 years, while an overwhelming 62% were discontinued in that time frame. These “leaks” in the clinical pipeline, where compounds with clinical potential are abandoned during clinical development, indicate that scientific innovations are not reaching the clinic and providing benefits to patients. This is true for not only novel candidates but also candidates from existing antibiotic classes with clinically validated targets. By identifying the sources of the leaks in the clinical pipeline, future developmental efforts can be directed toward strategies that are more likely to flow into clinical use. In this review, we conduct a detailed failure analysis of clinical candidates with Gram-negative activity that have fallen out of the clinical pipeline over the past decade. Although limited by incomplete data disclosure from companies engaging in antibiotic development, we attempt to distill the developmental challenges faced by each discontinued candidate. It is our hope that this insight can help de-risk antibiotic development and bring new, effective antibiotics to the clinic.

## INTRODUCTION

Bacterial resistance to antibiotics is a growing public health crisis; 1.27 million global deaths were attributed to multidrug resistance (MDR) in 2019 (1). Left unchecked, MDR could lead to 10 million global annual deaths in 2050 (2, 3). Modern medicine relies on antibiotics to control secondary bacterial infections from routine procedures like surgery and chemotherapy. These secondary infections may become untreatable due to antibiotic-resistant bacteria, escalating the risk of common medical procedures.

Of the most threatening MDR pathogens identified by the U.S. Centers for Disease Control and Prevention (CDC) (4) and the World Health Organization (WHO) (2), Gram-negative bacteria (GNB), including Klebsiella pneumoniae (of the *Enterobacteriaceae* family), Acinetobacter baumannii, and Pseudomonas aeruginosa, stand out as urgent unmet needs. In addition to their general intrinsic resistance to antibiotics, all three have developed critical resistance to the carbapenem class of antibiotics, leaving limited alternative treatment options (5, 6). Despite the growing threat of untreatable infections, the 2020 global antibiotic clinical pipeline contained only 23 candidates with GNB activity, none of which belonged to a new class (7). The high incidence of cross-resistance to existing antibiotics implies that the development of resistance to these new agents is closely trailing (8). While the success rate from phase 1 trials to U.S. Food and Drug Administration (FDA) approval for all antibacterial therapeutics between 2011 and 2020 was 16.3% (9), the last FDA-approved antibiotic with a novel mechanism of action against GNB was discovered nearly 60 years ago.

Clinical studies initiated in the 1980s and 1990s (largely cephalosporins, fluoroquinolones, and macrolides) had high success rates, with 40% of candidates obtaining market approval in a median time of 6 years (10). However, of the 61 antibiotics approved for use between 1980 and 2009, 43% have been withdrawn by the FDA, and the 6 antibiotics withdrawn due to safety issues were all fluoroquinolones (11). Moreover, the number of antibacterial investigational new drug (IND) applications filed with the FDA between 2010 and 2019 is the lowest that it has been in the past 4 decades (10). Despite the unique challenges of antibiotic discovery (12–15), 72% of candidates in the current global preclinical pipeline represent novel classes, with overlapping cellular targets and mechanisms of action that are distinct from those of antibiotics used in the clinic today (7, 16). The consequences of failure are unbearable for the small companies that drive antibiotic development and for the future of a society that so heavily depends on efficacious antibiotics.

Here, we profile antibiotic candidates with GNB activity that have fallen out of the clinical pipeline over the last decade and identify trends in their development. These vignettes are limited by the extent of information disclosure by the companies pursuing these candidates, but we hope to inform future discovery and development efforts by highlighting patterns in these failures. Stronger predictors of success may enable more diverse candidates from the preclinical pipeline to enter a de-risked clinical pipeline and emerge as FDA-approved therapeutics.

## OVERVIEW OF THE CLINICAL DEVELOPMENT PIPELINE FOR GNB-ACTIVE ANTIBIOTICS (2010 TO 2020)

**FIG 1:**
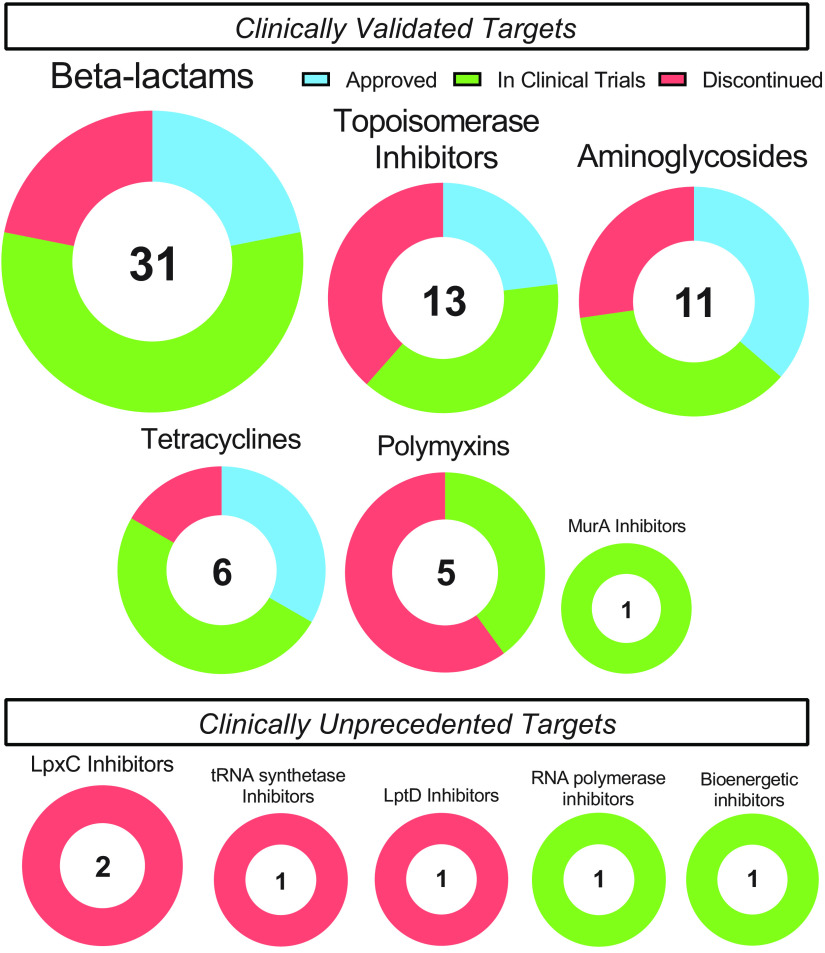
GNB-active clinical candidates by class and clinical trial status. Antibiotic classes that have undergone clinical development between 2010 and 2020 are represented as circles. Segments are colored according to the proportions of candidates in that class that have been approved, are currently in clinical development, or have been discontinued.

The clinical development pipeline for systemic GNB-active candidates over the past decade is detailed in Table 1. Despite the desperate need for antibiotics with novel targets and high target diversity in the preclinical pipeline, most candidates in clinical development are from clinically validated classes (Fig. 1), presumably due to the higher perceived risk of pursuing a non-clinically validated target. While half of all classes in development contain an antibiotic that has been approved in the past 10 years, the other half comprise unexploited antibiotic targets: tRNA synthetases, LpxC, and LptD.

**TABLE 1:**
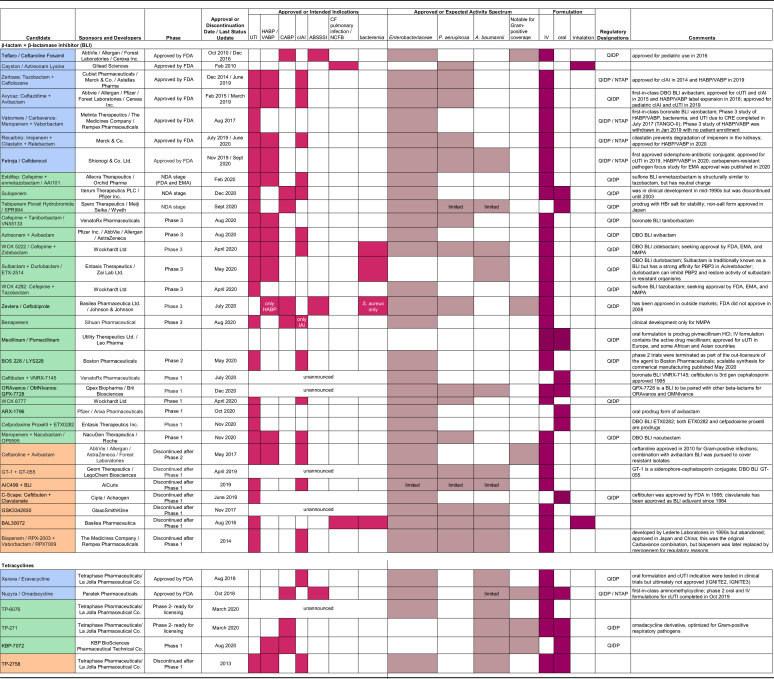

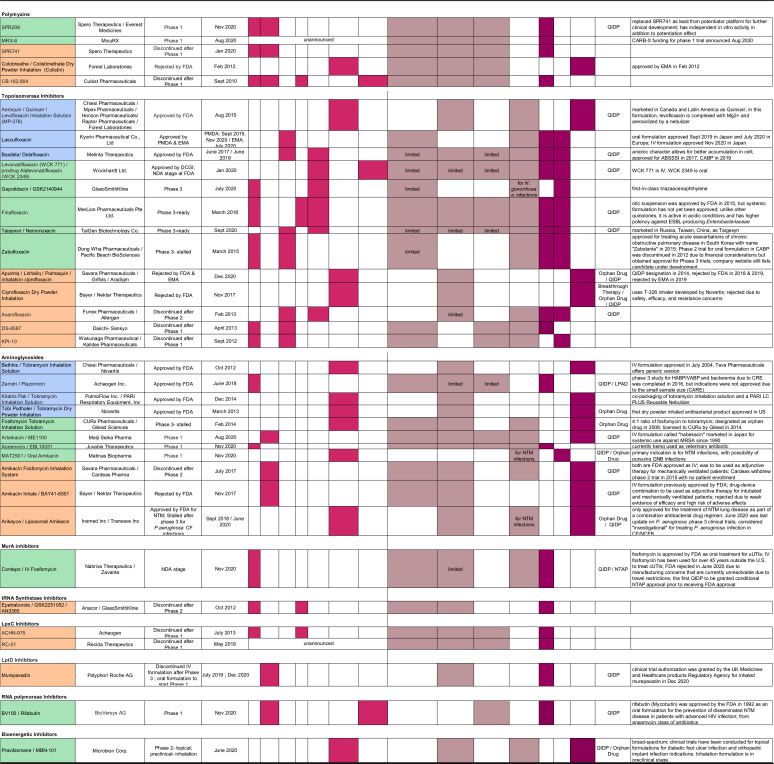
Clinical development details of GNB-active antibiotic candidates^*a*^

a In the first column, candidates that are approved are shaded blue, those that are in current development are shaded green, and those that are discontinued are shaded orange. In the columns for approved or intended indications, approved or expected activity spectrum, and formulation, a shaded cell indicates that the category shown in the subcolumn heading is a characteristic of the corresponding antibiotic candidate in the first column. New characteristics reported after the date listed in the column labelled last status update are not reflected in this table. DCGI, Drugs Controller General of India; EMA, European Medicines Agency; FDA, U.S. Food and Drug Administration; NDA, new-drug application, filed after clinical trials; NMPA, Chinese National Medical Products Administration; ABSSSI, acute bacterial skin and skin structure infection; CF, cystic fibrosis; cIAI- complicated intra-abdominal infection; HABP, hospital-acquired bacterial pneumonia; uUTI, uncomplicated urinary tract infection; NCFB, non-cystic fibrosis bronchiectasis; NTM, nontubercular mycobacterium; VABP, ventilator-associated bacterial pneumonia; IV, intravenous; Breakthrough therapy, FDA designation to expedite the development and review of drugs; LPAD, limited population pathway for antibacterial and antifungal drugs designation given by the FDA to indicate a limited-usage recommendation; NTAP, new-technology add-on payment designation given by the Centers for Medicare and Medicaid Services as an incentive for hospitals; Orphan Drug, FDA designation given as an incentive; QIDP, qualified infectious disease product designation given by the FDA as an incentive; CRE, carbapenem-resistant *Enterobacteriaceae*; DBO, diazabicyclooctane class of BLIs with PBP-binding properties; ESBL, extended-spectrum β-lactamase; PBP, penicillin-binding protein.

**FIG 2:**
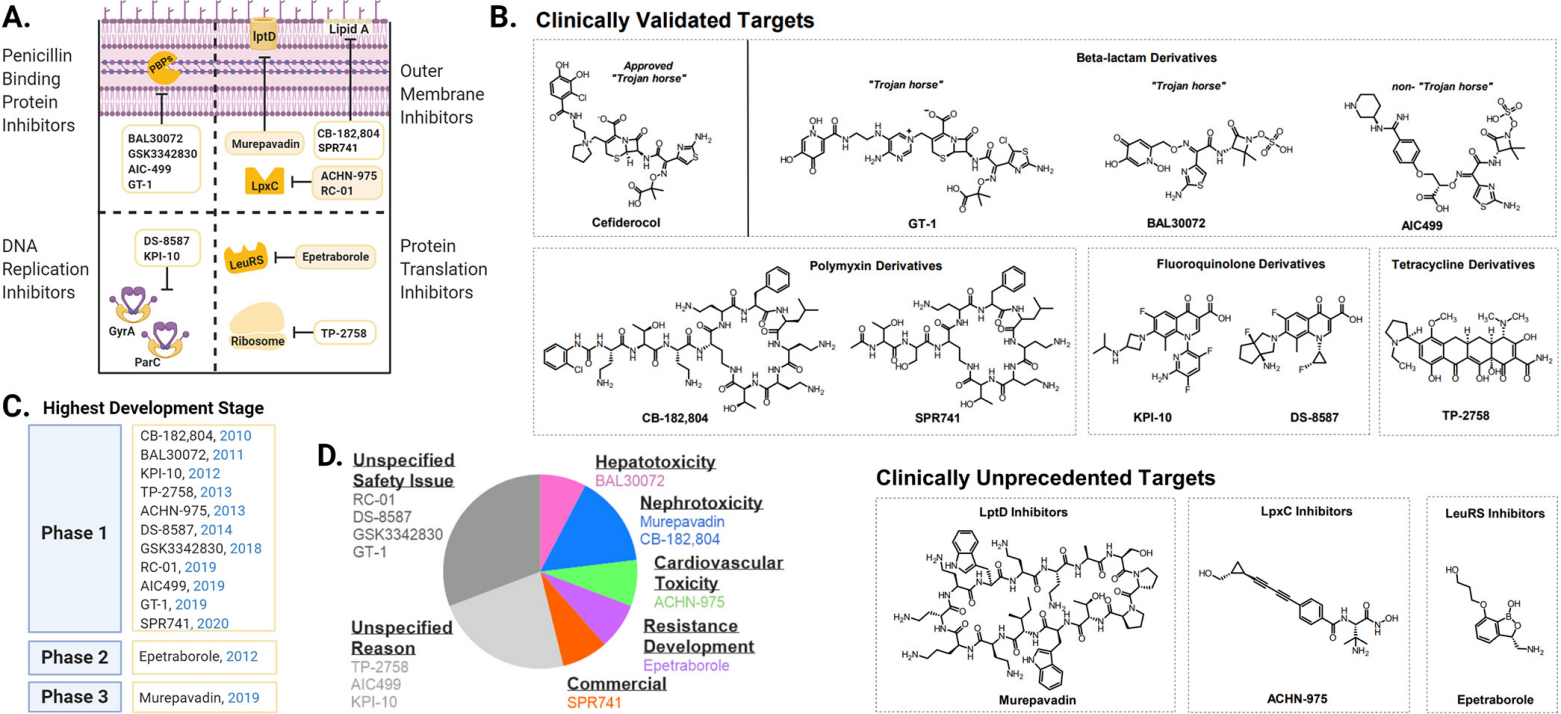
Profile of GNB-active clinical candidates discontinued between 2010 and 2020. (A) Cellular localizations of targets of discontinued candidates. Yellow boxes represent inhibitors of clinically unprecedented targets. (B) Chemical structures of discontinued candidates. Structures were retrieved from the following PubChem identifiers: 91824766 for murepavadin, 71466517 for ACHN-975, 405560444 for CB-182,804, 53323381 for SPR740, 135905457 for BAL30072, 56640741 for DS-8587, 11676981 for KPI-10, 46836890 for epetraborole, and 77843966 for cefiderocol. The TP-2758 structure was not found in PubChem and was instead replicated from the structure described previously by Sun et al. (49). The AIC499 structure was not found in PubChem and was instead replicated from the structure described previously by Freischem et al. (39). Structures of RC-01 and GSK3342830 are not disclosed. (C) Year and clinical trial stage at the time of discontinuation. Candidates appear in chronological order for each trial stage. (D) Reasons for discontinuation. Limited information was gathered from public press releases and published literature. (Created with BioRender.com.).

Although most discontinued candidates are first-time entrants into the clinical development pipeline, some candidates have traversed the pipeline as a different formulation (for example, inhalation therapies) or purposed for other indications (for example, label expansions). The remainder of this review profiles the journey of the 13 first-time entrants that have fallen out of the clinical pipeline. These select candidates target components of the outer membrane (OM), DNA replication, protein translation, and penicillin-binding proteins (PBPs) (Fig. 2A). The structural diversity (Fig. 2B) reflects the variety of mechanisms of action employed to inhibit GNB growth. Most of these candidates were discontinued after phase 1 (Fig. 2C) due to safety concerns (Fig. 2D).

## DISCONTINUED CANDIDATES WITH CLINICALLY VALIDATED TARGETS

### β-Lactam derivatives.

The degradation of β-lactams by β-lactamases is a common resistance mechanism that has been partially addressed by structural optimization of the β-lactam scaffold, adjunctive administration of β-lactamase inhibitors (BLIs), and attachment of a siderophore for improved cellular uptake (17). Among the many attempts since 1980 to overcome resistance by attaching an iron-chelating group to a β-lactam (18, 19), cefiderocol was the first siderophore-antibiotic conjugate to gain FDA approval in 2019. No other clinical-stage siderophore–β-lactam conjugate (cefetecol, BAL30072, GSK3342830, and GT-1) has progressed past phase 1 trials.

### (i) BAL30072.

BAL30072 is a siderophore-monobactam conjugate developed by Basilea Pharmaceutica (Basel, Switzerland) derived from tigemonam, with an appended dihydroxypyridinone moiety for iron chelation. Portions of the structure resemble those of aztreonam and avibactam. BAL30072 exhibits bactericidal activity against P. aeruginosa, Acinetobacter species, and *Enterobacteriaceae* and is stable to metallo-β-lactamases (20, 21). While most monobactams singularly inhibit PBP3, BAL30072 also engages the bifunctional PBP1a and -1b in Escherichia coli (20). Accordingly, while filamentation is usually observed in E. coli cells treated with monobactams targeting PBP3 (22), BAL30072 triggers spheroplasting prior to lysis (20). This spheroplasting phenotype is also elicited by some bicyclic β-lactams (23) and β-lactamase enhancers that target PBP2 (24).

Several *in vitro* studies indicate the synergy of BAL30072 in combination with meropenem or colistin against various MDR GNB clinical isolates (25–27). *In vivo* synergy was evaluated in soft tissue infection models of rats challenged with A. baumannii: while BAL30072 showed statistically significant activity, the addition of meropenem was not additive, synergistic, or antagonistic (25). This finding may be rationalized: both BAL30072 and carbapenems inhibit PBP2 in A. baumannii, limiting the pair’s success to mere additive effects. The synergy of these antibiotics might be exploited against *Enterobacteriaceae* or P. aeruginosa, where they have complementary PBP-binding profiles (27). In murine septicemia, the combination therapy offered protection against carbapenem-resistant P. aeruginosa and MDR A. baumannii, the former due to complementary PBP-binding profiles and the latter possibly due to complementary β-lactamase-binding profiles (27).

A 2010 phase 1 single-ascending-dose (SAD) study reported no serious adverse events at doses of up to 8 g. The multiple-ascending-dose (MAD) study established a maximum tolerated dose, limited by elevated alanine aminotransferase (ALT) enzyme levels (a measure of liver injury). In 2014, Basilea initiated another phase 1 MAD study of BAL30072, both alone and in combination with meropenem. When 2 g BAL30072 was administered as 1-h intravenous (i.v.) infusions every 8 h (6 g/day) or when 4 g of BAL30072 was administered as continuous 22-h infusions for 6 days, abnormally high ALT levels were observed in almost all healthy study subjects by as early as 3 days posttreatment, and the development of the i.v. formulation was ceased (28). *In vitro* studies revealed that BAL30072 inhibits the mitochondrial electron transport chain, β-oxidation, and glycolysis in HepG2 liver cells at concentrations of 100 to 200 μM, which is clinically relevant only after long-term exposure (28, 29). These findings were unexpected given positive toxicity studies in rats and marmosets dosed with BAL30072 for 4 weeks (28).

To assess utility for urinary tract infection (UTI), urinary concentrations of BAL30072 were analyzed in MAD study subjects (27, 29). Bactericidal activity against P. aeruginosa was weak in urine, presumably due to a low concentration of iron and consequent competition with native siderophores (29). Basilea also began preclinical studies of an inhalation formulation for the treatment of pulmonary infections in cystic fibrosis (CF) patients, which was stopped in 2016 due to a lack of confidence in the candidate’s success (30).

### (ii) GSK3342830.

GlaxoSmithKline (GSK) (London, UK) and Shionogi (Osama, Japan) initiated a collaboration in 2010 to discover novel cephem antibiotics with GNB activity, yielding two promising cephalosporin-siderophore conjugates. In 2015, Shionogi retained rights to cefiderocol, which became the first siderophore-antibiotic conjugate to gain FDA approval (31), and GlaxoSmithKline retained rights to the catechol-cephem GSK3342830.

Phase 1 GSK3342830 trials began in 2017 (32). In the SAD component, pharmacokinetic (PK) properties were consistent with those of other cephalosporins, including cefiderocol, and no severe adverse events were detected at doses of up to 6 g (33). In the MAD study, 11 subjects received 1 g GSK3342830 as a single i.v. infusion on day 1, 3-times-a-day i.v. infusions on days 2 through 14, and a single i.v. infusion on day 15. Four participants discontinued the treatment due to headache, malaise, and/or fever, and 1 had high ALT levels leading to automatic discontinuation. The 6 subjects remaining in the study experienced malaise, headache, and fever with onset at between 9 and 10 days and a general decrease in platelet counts (33). While symptoms could be related to known off-target binding to the 5HT-3 serotonin receptor, this interaction seemed physiologically unlikely (33). GSK3342830 was discontinued following these results in 2018.

### (iii) GT-1 and GT-055.

GT-1 (LCB10-0200) is a siderophore-cephalosporin conjugate developed by LegoChem Biosciences (Daejeon, South Korea) in a joint venture with Geom Therapeutics (San Francisco, CA, USA). The candidate features the same dihydroxypyridinone siderophore appendage as the one present in BAL30072 and a side chain similar to that of ceftazidime. GT-1 demonstrated efficacy against P. aeruginosa in murine models of systemic, thigh, respiratory tract, and urinary tract infections (34). Its activity spectrum also covers MDR *Enterobacteriaceae* and A. baumannii (35). The candidate was paired with GT-055 (LCB18-055), a diazabicyclooctane BLI with intrinsic activity against PBP2 (36, 37).

A phase 1 study was registered in Australia in 2019 (38). Only 8 participants were enrolled in this trial when it was terminated due to unspecified safety reasons, presumably hepatotoxicity.

### (iv) AIC499.

AIC499 is a monobactam bearing high resemblance to aztreonam with notable activity against MDR A. baumannii and P. aeruginosa. Structural analysis shows hydrophobic interactions between the phenyl portion of the head group and PBP3, while the piperidine portion has a dynamic configuration with a lesser impact on binding yet beneficial PK/pharmacodynamic (PD) properties (39). The candidate was noted to have potent antibacterial activity when coadministered with a BLI, although the combination that AiCuris Anti-infective Cures GmbH (Wuppertal, Germany) pursued in clinical trials was unspecified. Phase 1 began in Austria in 2017, with phase 2 planned for complicated intra-abdominal infection (cIAI) and complicated urinary tract infection (cUTI). These results are unpublished, and the candidate was removed from the company’s pipeline in 2019 for undisclosed reasons.

### Fluoroquinolone derivatives.

Fluoroquinolones began receiving FDA approval in the late 1960s for treating UTIs and respiratory tract infections, but the FDA has issued many side-effect warnings for these antibiotics since 2008. Reports of these adverse events during postmarketing surveillance led to the withdrawal of several fluoroquinolones. Second- and third-generation fluoroquinolones like ciprofloxacin, levofloxacin, and moxifloxacin are still used to treat GNB infections.

### (i) DS-8587.

DS-8587 is a broad-spectrum fluoroquinolone synthesized by Daiichi Sankyo (Tokyo, Japan) with enhanced bactericidal activity against Acinetobacter baumannii. The candidate retains the core structure of post-second-generation fluoroquinolones, most closely resembling moxifloxacin; however, the fluorination of the cyclopropyl group, the C_7_ octahydrocyclopentapyrrole, and the methylated C_8_ distinguish the candidate from the newer-generation candidates that have other fused pyrrolidines at C_7_ and an ether or no functionality at C_8_. The dual-targeting compound has micromolar IC50 (50% inhibitory concentration) values for the A. baumannii ParC and GyrA enzymes, high potency against clinical isolates of A. baumannii with mutated ParC and GyrA domains, and low resistance frequency and efflux pump susceptibility (40). In murine calf muscle infection, efficacy was correlated with area under the concentration-versus-time curve (AUC)/MIC values, like other quinolones (41).

Daiichi Sankyo previously marketed three fluoroquinolones (ofloxacin, levofloxacin, and sitafloxacin), but DS-8587 development was discontinued in 2014 after phase 1 for unexplained reasons. Studies from 2017 revealed the *in vivo* efficacy of DS-8587 against Fusobacterium necrophorum, a pathogenic obligate GNB anaerobe, in murine liver abscess (42).

### (ii) KPI-10.

KPI-10 (WQ3813) is a synthetic fluoroquinolone, bearing similarity to 4th-generation trovafloxacin, discovered by Wakunaga Pharmaceutical (Osaka, Japan). The broad-spectrum activity against *Enterobacteriaceae*, MDR Acinetobacter species, Neisseria gonorrhoeae, and notable Gram-positive organisms, including methicillin-resistant Staphylococcus aureus (MRSA) and Streptococcus pneumoniae (43–45), pointed toward the candidate’s utility in treating both community-acquired bacterial pneumonia (CABP) and UTI.

Kalidex Pharmaceuticals (Menlo Park, CA, USA) licensed the global development and commercialization rights to the candidate. Phase 1 of the oral formulation began in 2012. The SAD study demonstrated a favorable safety and PK profile, supporting a daily oral dosing regimen (46). Clinical development was discontinued for undisclosed reasons, and Kalidex reportedly ceased operation in 2016.

### Tetracycline derivatives: (i) TP-2758.

Tetraphase (Watertown, MA, USA) optimized the convergent total synthesis of tetracycline to access analogs that are inaccessible by semisynthesis (47). This approach produced one clinically approved antibiotic (eravacycline) and two other phase 1 candidates (TP-271 and TP-6076). TP-2758, with a chiral 8-pyrrolidinyl substitution, was discovered while generating a series of novel 7-methoxy-8-heterocyclyl tetracycline analogs (48). Derivatives of tetracyclines, called glycylcyclines, were developed to combat the rise of tetracycline resistance. While most tetracyclines are orally dosed, glycylcyclines like tigecycline are restricted to i.v. dosing. TP-2758 was projected to become the first orally bioavailable glycycline.

TP-2758 was more potent than tigecycline against A. baumannii and *Enterobacteriaceae*, and both oral and i.v. dosing of TP-2758 significantly reduced the burden of infection in murine pyelonephritis induced by E. coli or MDR K. pneumoniae (49). Oral bioavailability values vary between animal species: while tetracycline has oral bioavailabilities of only 14.9% in rats and 6.7% in monkeys, it is >70% in humans (49). TP-2758 had oral bioavailabilities of 8.62% in rats and 30.4% in monkeys, implying higher oral bioavailability in humans than tetracycline (49). Phase 1 studies (50) for the oral formulation began in 2011, but results are unavailable. TP-2758 was removed from the company’s pipeline in 2013, and Tetraphase was acquired by La Jolla Pharmaceutical Company in 2020.

### Polymyxin derivatives.

Polymyxins are cationic cyclic peptides (net charge of +5) thought to selectively disrupt and permeabilize the GNB OM to result in bactericidality, although evidence suggests that they may have more than one target (51). When polymyxins were first introduced to the clinic, they were quickly abandoned due to high incidences of dose-limiting nephrotoxicity and neurotoxicity (52). However, with the rise of MDR Gram-negative pathogens, this class has resurged in the clinic as a last-resort therapy (53). The two clinically administered polymyxins, polymyxin B (PMB) and colistin, are manufactured by fermentation as an impure, heterogeneous mix of related compounds. CB-182,804 was the first polymyxin to undergo clinical trials under the FDA’s oversight.

### (i) CB-182,804.

BioSource Pharmaceuticals (Spring Valley, NY, USA) developed a semisynthetic route to substitute the N-terminal fatty acyl group that contributes to the toxicity of PMB by utilizing a deacylase enzyme from the microorganism Actinoplanes utahensis (54). After screening many urea-linked halophenyl functionalities for antimicrobial activity, the 2-chlorophenylurea derivative CB-182,804 emerged as a lead candidate. The candidate had bactericidal activity against E. coli, K. pneumoniae, P. aeruginosa, and A. baumannii. Cubist Pharmaceuticals (Lexington, MA, USA) obtained a provisional license for the candidate, and subsequent patents were filed jointly to further develop the strategy (55).

The MICs of CB-182,804 against 5,000 clinical isolates were only 2-fold higher than those of PMB, with observable cross-resistance (56). Similarly, *in vivo* efficacies in murine P. aeruginosa lung and A. baumannii thigh infection models were comparable for the two (57). However, the 50% effective concentration (EC_50_) values against a rat renal tubule cell line were >1,000 mg/L for CB-182,804 and 318 mg/L for PMB (57). In cynomolgus monkeys dosed at 6.6 mg/kg of body weight/day 3 times a day for 7 days, CB-182,804 showed limited renal tubular histological changes, whereas PMB exhibited renal tubular degeneration; at a higher dose of 9.9 mg/kg/day, CB-182,804 elicited only slight increases in blood urea nitrogen and serum creatinine, whereas PMB elicited severe signs of nephrotoxicity (58). CB-182,804 also demonstrated more favorable PK/PD parameters than PMB, including decreased serum protein binding, increased plasma clearance, increased volume of distribution, less systemic exposure, as well as a lower maximum concentration of drug in serum (*C*_max_) (58).

Clinical trials began in February 2009, but the development of this molecule ceased in 2010, presumably due to nephrotoxicity issues (59). Cubist was acquired by Merck Pharmaceuticals in 2015.

### (ii) SPR741.

SPR741 (NAB741) is a fully synthetic PMB derivative that was designed to curtail nephrotoxicity issues associated with this class through a reduced positive charge (3+) and the removal of the highly lipophilic fatty acid side chain in PMB (60). In a rat model, renal clearance of SPR741 was 400-fold higher than that of colistin, suggesting improved safety-related PK properties (60). Despite having weak antibacterial activity, sub-MIC dosing of SPR741 enhances the permeation of other antibiotics through the OM (61). *In vivo* studies confirm this potentiation with expanded azithromycin coverage against MDR *Enterobacteriaceae* (62) and synergy with rifampicin against extremely drug-resistant (XDR) A. baumannii (63).

In a phase 1 drug-drug interaction study, i.v. dosing of other antibiotics (1.0 g of ceftazidime, 4.5 g of piperacillin-tazobactam, or 1.0 g of aztreonam) with 400 mg of SPR741 did not significantly affect the concentration-versus-time profile, clearance, or half-life of either drug (64). In the MAD study, 25% of subjects experienced decreased creatinine clearance across all drug dosage cohorts: 3 in the 600-mg, 1 in the 400-mg, 1 in the 150-mg, and 1 in the 50-mg cohorts (64). Of these 6 subjects, 5 had normal creatinine levels at day 16, while 1 from the 600-mg cohort had a moderate increase in the serum creatinine level above the baseline level that began on day 14. SPR741 was discontinued in January 2020 and replaced by SPR206, a different polymyxin analog from the potentiator platform. While SPR741 was developed as an antibiotic adjuvant, SPR206 has antibacterial activity as a standalone therapy and boasts potentially superior safety and efficacy profiles to those of SPR741.

## DISCONTINUED CANDIDATES WITH CLINICALLY UNPRECEDENTED TARGETS

### Murepavadin (LptD inhibitor).

Inspired by the antimicrobial host defense peptide protegrin I, Polyphor Ltd. (Allschwil, Switzerland) synthesized and screened a library of β-hairpin-shaped macrocyclic protein epitope mimetics for antimicrobial activity (65–68). While initial leads exhibited hemolysis of red blood cells and degradation by serum enzymes, optimization toward antibacterial activity yielded the clinical candidate murepavadin (POL-7080) (68, 69). Murepavadin reportedly targets the β-barrel protein LptD (69–71), an essential (72) surface-exposed OM protein that acts in a complex (73–75) to incorporate lipopolysaccharide (LPS) into the OM of GNB. The differential N-terminal lengths of LptD among GNB is thought to confer the specificity of murepavadin to the P. aeruginosa protein (69). In preclinical studies, murepavadin outperformed comparator antibiotics, including colistin, against even XDR P. aeruginosa clinical isolates (76, 77). Although oral bioavailability was low in rats, subcutaneous administration in humans yielded a bioavailability of 67 to 79% and a half-life of 5 to 8 h. The discovery and development of murepavadin has previously been reviewed (78).

Roche (Basel, Switzerland) obtained a license to develop and commercialize murepavadin in 2013. Six phase 1 studies explored the safety, tolerability, and PK of murepavadin: a combined SAD and MAD study in healthy male subjects (79), a multiple-dose study evaluating the penetration of murepavadin into the lungs (80), a drug-drug interaction investigation of murepavadin with colistin (81) and with amikacin (82), a thorough QT (TQT) (in reference to QT intervals measured by an electrocardiogram) study with SAD (83), and an SAD study of murepavadin in subjects with renal function impairment (84). Systemic exposure to murepavadin was increased in subjects with renal function impairment, indicating a need for dose adjustment based on the creatinine clearance rate (85). Despite Roche returning the murepavadin development license to Polyphor in 2015, two phase 2 studies were successfully completed: a 14-day dosage of murepavadin in subjects with acute exacerbation of non-cystic fibrosis bronchiectasis due to P. aeruginosa infection (86) and a MAD study of murepavadin coadministered with the standard of care (SOC) in subjects with ventilator-associated bacterial pneumonia (VABP) due to P. aeruginosa infection (87). In the latter study, clinical cure was achieved in 10 out of 12 (83%) patients with confirmed P. aeruginosa infection, and the 28-day all-cause mortality rate in this population was 9% (88).

Although murepavadin’s narrow spectrum of activity provides advantages as a treatment option, it complicated the phase 3 clinical trial design (89). While phases 1 and 2 tested murepavadin as a monotherapy, the ethics of phase 3 trials in pneumonia patients necessitated the coadministration of murepavadin with a broad-spectrum drug (89). The coadministered antibiotic needed to have no pseudomonal activity to avoid confounding the results of the trial. Ertapenem, a first-line therapy for CABP, was ultimately chosen for coadministration, and the appropriate dosing for hospital-acquired bacterial pneumonia (HABP)/VABP was determined (89).

Murepavadin underwent two separate phase 3 trials to test its efficacy in HABP/VABP infection due to P. aeruginosa (90, 91). The FDA-approved noninferiority study (PRISM-UDR) (90) compared murepavadin plus ertapenem to 1 β-lactam antibiotic to treat HABP/VABP driven by P. aeruginosa in clinical centers with a low incidence of MDR. The European Medicines Agency (EMA)-approved study (PRISM-MDR) (91), in contrast, compared murepavadin plus 1 antipseudomonal antibiotic to 2 antipseudomonal antibiotics in clinical centers with a high incidence of MDR to assess murepavadin efficacy over the SOC. Although a 25 to 40% incidence of kidney injury was anticipated based on the comparator arm, 56% of patients treated with murepavadin in the VABP study showed evidence of acute kidney injury (92). Polyphor terminated i.v. formulation development as of July 2019 due to nephrotoxicity concerns. Murepavadin was the only GNB-active clinical candidate in this decade to be discontinued after phase 3. Polyphor continued the preclinical development of an inhalation formulation of murepavadin, and clinical trial authorization was granted in the United Kingdom in December 2020.

### ACHN-975 (LpxC inhibitor).

LpxC is a cytosolic zinc-dependent metalloenzyme that catalyzes the first committed step of lipid A biosynthesis. While many antibiotic discovery programs have pursued LpxC inhibitors (93), Achaogen’s (South San Francisco, CA, USA) structure-based discovery effort yielded the first LpxC inhibitor to advance into clinical trials. Like other previously patented LpxC inhibitors (94, 95), this synthetic compound contains a hydroxamic acid moiety that coordinates the catalytic Zn^2+^ and a long hydrophobic tail that interacts with the active-site tunnel.

While the genetic sequence of LpxC is highly conserved across GNB, the subtle structural differences in LpxC influence the potency and dynamics of inhibition (96). ACHN-975 exhibited optimal efficacy when the dose was administered once daily for P. aeruginosa but administered multiple times a day for E. coli and K. pneumoniae, so an intermittent high-dose regimen was established to treat respiratory P. aeruginosa infections (97). The possibility of resistance emergence set the minimum required dose: at concentrations 4-fold higher than the MIC, the frequency of resistance ranged from 10^−7^ to 10^−10^ in P. aeruginosa clinical isolates (97). However, ACHN-975 induces bradycardia in preclinical animal models (98), setting a maximum tolerated dose.

In 2012, a phase 1 SAD study to assess the candidate’s safety, tolerability, and PK in 50 healthy volunteers (99) was completed. The therapeutic window was deemed insufficient due to concentration-driven dose-limiting cardiovascular toxicity (transient hypotension without tachycardia), which occurred in the first subject who received an 18-mg/kg infusion (100). A 2013 MAD study (101) was prematurely terminated after enrolling four subjects. Participants encountered inflammation at the infusion site after repeat dosing of 4 mg/kg three times a day for 3 to 4 days.

In 2015, Achaogen began an optimization program focusing on P. aeruginosa (100). This pathogen was more sensitive to LpxC inhibition in *in vivo* models than *Enterobacteriaceae* species, and the structural features of P. aeruginosa LpxC seemed more amenable to curtailing drug toxicity (100). To investigate structure-toxicity relationships, a high-content assay in anesthetized rats was developed to assess maximum tolerated concentrations (100). Cardiovascular toxicity was attributed to a nonspecific effect of basic amines, so a new candidate was identified with a wider therapeutic window. With the removal of the amine, this new candidate was nonsolubilizable at 10- to 100-mg/mL concentrations using acidic pH (100). To overcome solubility issues and accommodate the anticipated dose of >1 g per day, the hydroxyl tail was converted to a phosphate prodrug. Surprisingly, this new prodrug, dosed in a simple aqueous formulation, demonstrated cardiovascular toxicity in the anesthetized rat model, even though the parent molecule, dosed in pH-adjusted hydroxypropyl-cyclodextrin, did not (100). Compounds and insights from these studies were passed on to Forge Therapeutics (San Diego, CA, USA) after Achaogen filed for bankruptcy in 2019.

### RC-01 (LpxC inhibitor).

Fujifilm Toyama Chemical Co. Ltd. (Toyoma, Japan) screened compounds with malonamide, a derivative of the zinc-chelating hydroxamic acid, for LpxC activity. RC-01 (T-1228) was identified as a lead compound, exhibiting a subnanomolar IC_50_ against LpxC and bactericidal activity against P. aeruginosa and *Enterobacteriaceae* (102). *In vitro* exposure of RC-01 to GNB reduces the release of LPS (103), corroborating *in vivo* data from other LpxC inhibitors that decrease LPS-dependent stimulation of the host immune system, thereby attenuating bacterial virulence (104). In mouse models of P. aeruginosa-induced pneumonia and E. coli-induced UTI, the PK/PD parameter most highly correlated with efficacy was the AUC for the free, unbound fraction of the drug (*f*AUC)/MIC ratio (105). The frequency of resistance to RC-01 at 4× MIC was 10^−7^ to 10^−8^ (106).

In 2019, Recida Therapeutics (Menlo Park, CA, USA) licensed the development and commercialization rights for RC-01 outside Japan. LpxC-associated cardiovascular toxicity was unapparent with RC-01: at least 400 mg/kg/day was tolerated in 2-week repeated i.v. dosing in rats and dogs, with unreported *f*AUC and *C*_max_ (107). Two formulations of RC-01 were pursued: an inhalation therapy for respiratory infections and i.v. therapy for systemic infections. The programs were prematurely terminated after enrolling 8 subjects in a phase 1 SAD study (108) for unspecified safety reasons. Recida soon after surrendered its business rights in California, and MicuRx was granted rights for investigational treatment with RC-01 in China.

### Epetraborole (LeuRS inhibitor).

Epetraborole (GSK2251052; AN3365) is a bacteriostatic oxaborole-containing inhibitor (109) of leucyl-tRNA synthetase (LeuRS) that was discovered in a structure-based rational design screen led by Anacor Pharmaceuticals (Palo Alto, CA, USA). The only FDA-approved aminoacyl-tRNA synthetase inhibitor is mupirocin, which targets isoleucyl-tRNA synthetase for the treatment of Gram-positive infections (110–112). Mupirocin is restricted to topical use due to the rapid metabolism of its ester moiety and resistance emergence (113).

The mechanism of a benzoxaborole antifungal agent trapping the active conformation of the editing site of LeuRS inspired the rational design of epetraborole (114). Guided by crystallography, benzoxaborole analogs with extended coverage against A. baumannii were synthesized (114, 115). Screening against MDR clinical isolates demonstrated a 10^−7^ one-step resistance frequency at 4× MIC (115), coverage of anaerobic microorganisms (116, 117), and low MIC_90_s against P. aeruginosa (118). Mouse thigh infections highlighted the candidate’s efficacy against MDR GNB *in vivo* (115).

In 2009, Anacor initiated phase 1 trials for the i.v. formulation and reported favorable safety and PK properties in 72 subjects (119). In accordance with a 2007 alliance forged with GlaxoSmithKline (London, UK), GSK obtained an exclusive license for epetraborole in 2010. Phase 1 trials included SAD and MAD studies of oral formulations (120), a small-cohort mass balance study of the i.v. formulation (121), and serum and pulmonary PK of the i.v. formulation (122). Like mupirocin, epetraborole is highly metabolized in monkeys and humans: the oxidation of the propanol side chain by the polymorphic alcohol dehydrogenase generates an inactive carboxylic acid metabolite (123). Following a 1,500-mg i.v. infusion of the candidate in 6 human subjects, the candidate was found in systemic circulation and urinary excretions in its original form and, to a great extent, its oxidized form.

GSK initiated phase 2 trials for cUTI (124) and cIAI (125). In 3 of the 14 patients receiving epetraborole in the cUTI study, resistant isolates were recovered after only 1 day of treatment (126). Whole-genome sequencing revealed target-specific mutations in the LeuRS editing domain that conferred a low fitness cost (126). The emergence of these fit mutants suggests that either this specific mode of binding to LeuRS or general inhibition of LeuRS is unproductive for impeding bacterial growth. Due to resistance concerns, the cUTI study was terminated in 2012, and the cIAI study was terminated as a precaution, even though isolates from 3 of the 9 patients who received epetraborole in this study maintained baseline susceptibility to the drug candidate (126). GSK also assessed drug distributions in epithelial lining fluid and alveolar macrophages, which showed promise for efficacy under a pneumonia indication (127). GSK soon after returned licensing rights to Anacor, which was acquired by Pfizer in 2016.

## DISCUSSION

A decade of leakiness in the GNB-active antibiotic clinical development pipeline is apparent from this review. The most prominent crack in the pipeline is the transition between phase 1 and phase 2. Data from AntibioticDB (128), a growing repository for antibiotics in global preclinical and clinical development from the 1960s to the present, show similar termination frequencies by clinical stage of development. In contrast, drugs from other therapeutic areas (including the “infectious disease” category) have the lowest success rate in the transition from phase 2 to phase 3 trials (129).

Both AntibioticDB and Hay et al. cite toxicology concerns (observable in phase 1) and lack of efficacy (after phase 1) as equally large determinants of failure for clinical candidates with disclosed discontinuation reasons. For the GNB-active candidates of this decade, however, halts over the past 10 years are largely attributable to safety issues in phase 1 trials; besides safety, three candidates were discontinued for unknown reasons, only one encountered resistance, one was replaced officially for commercial reasons, and none cited efficacy concerns (Fig. 2C).

Of the 13 discontinued candidates, 4 could have been first-in-class inhibitors, representing 3 novel targets: LptD, LpxC, and LeuRS. CB-182,804 was the first polymyxin to undergo clinical trials. Three of the four discontinued β-lactams attempted to follow the siderophore-antibiotic conjugation strategy successfully employed for cefiderocol. Overall, it is unclear whether novel targets are exceptionally failure prone given their small sample size. The poor safety profiles of these novel candidates may be due to the modalities of inhibiting new targets and/or the unanticipated toxicities of the novel chemical scaffolds. In the search for new antibiotics, the termination of first-in-class antibiotics is especially painful, as these new drugs provide hope for evading MDR.

Some of these discontinued clinical candidates do not strictly follow empirical guidelines for antibiotic design (130, 131). For example, while epetraborole was the only candidate terminated due to the emergence of resistance, the LpxC inhibitors ACHN-975 and RC-01 posed the same concerns for resistance due to their requisite high exposure and single-copy-single-enzyme-targeting mechanism (97). Additionally, ACHN-975 chelates the catalytic zinc of LpxC with hydroxamic acid, which is associated with the release of toxic metabolic by-products and off-target inhibition (100, 132–134). However, replacing the moiety impairs inhibitory potency and antibacterial activity with persisting toxicity (135, 136), underscoring the need for probing structure-toxicity relationships in new antibiotic classes. Conceivably, *in vivo* preclinical models are good predictors of antibacterial efficacy but poor predictors of safety, and alternate methods for assessing structure-toxicity relationships *in vitro* and *in vivo* should be developed.

The termination of some candidates was surprising considering the published toxicity data. Although hepatotoxicity was unapparent in preclinical models, BAL30072 treatment caused elevated ALT levels after only 3 days. *In vitro* nephrotoxicity is an unreliable predictor of clinical nephrotoxicity (137), which is especially problematic for polymyxins like CB-182,804 (138, 139). Despite decades of polymyxin use, structure-toxicity relationships of this class are still understudied; this gap in understanding coupled with the characteristic toxicity of this class may account for the dearth of analog development (140). Likewise, the long history of the β-lactam class, the similarity of siderophore-conjugated candidates to approved antibiotics, and the prior approval of one siderophore-conjugated antibiotic were insufficient to bring more siderophore-conjugated antibiotics to the clinic, and a better understanding of structure-toxicity relationships of the linker and iron chelator components may de-risk future development. The case of murepavadin highlights a latent nephrotoxicity concern that surfaced only in phase 3: phase 1 and 2 trials comprised 8 studies, in which 257 subjects received at least a single dose of murepavadin for up to 15 days, and the only 3 serious adverse events (SAEs) reported were fully reversible after discontinuation (78). As patients in phase 3 trials are typically sicker than the healthy subjects in phase 1, antibiotic toxicology must account for higher-acuity settings.

Can discontinued candidates be revived in the clinical pipeline? Polyphor has already initiated murepavadin clinical development by reformulating from i.v. to oral. An inhalation formulation could benefit pneumonia treatment candidates with dose-limiting toxicity by decreasing systemic exposure and increasing concentrations in lung tissues (141), and all approved inhaled antibiotics are reformulations of compounds initially dosed through the i.v. or oral route.

Another strategy for candidates with dose-limiting toxicity is coadministration in a synergistic combination therapy to expand their therapeutic window. While this strategy invites challenges pertaining to matching PK properties, it has been employed for several antibiotics: novel BLIs have successfully extended the spectrum of β-lactams to MDR GNB (142). In addition to binding β-lactamases, some potentiators inhibit cell growth by PBP-binding-dependent and -independent mechanisms (143, 144). Discontinued PBP-binding candidates could be explored further in combination with a BLI or as an adjuvant for other β-lactams of complementary PBP-binding and β-lactamase-binding properties. For example, the synergy of BAL30072 with meropenem compelled Basilea to pursue combination therapy in phase 1 trials despite the dose-limiting hepatotoxicity encountered in the previous MAD study of BAL30072 alone.

Similarly, antibiotic potency and/or spectrum of activity can be potentiated with polymyxins. Polymyxins have been investigated as potentiators for other classes of antibiotics without conclusive evidence of synergy in clinical treatments (145, 146). *In vitro* studies show evidence of polymyxin synergy with many antibiotics (147), including the addition of rifampicin to CB-182804 to improve potency and MDR coverage (56). SPR741 employed this potentiation strategy, although it was discontinued after phase 1 trials for commercial reasons.

Additionally, LpxC inhibitors have demonstrated synergy with antibiotics for which GNB activity is limited by the OM, like rifampicin and tetracycline (93). LpxC inhibition may contribute to A. baumannii clearance *in vivo* by enhancing bacterial opsonophagocytosis and reducing inflammation (104) despite the nonessentiality of LPS biosynthesis in this species and the resultant *in vitro* inefficacy of LpxC inhibitors. This antivirulence-based mechanism of action may reduce its likelihood of encountering resistance and extend the coadministered antibiotic’s spectrum of activity.

Finally, there is a critical yet latent misalignment of the antibiotic discovery pipeline with the clinical development pipeline (148). While antibiotic discovery typically focuses on identifying candidates corresponding to a particular MDR pathogen, cellular target, or chemical structure, late-stage clinical trials primarily test the candidate’s efficacy in the context of clinical indications. Even if a candidate fills an unmet need by targeting a critical MDR pathogen or demonstrating low cross-resistance, that coverage may be moot when tested at clinical trial sites with low incidences of MDR and compared to SOCs with high efficacy against susceptible pathogens (149). Since the rapid determination of an infection’s causative organism is usually infeasible, empirical treatment based on infection site is common. Recently, the FDA required an infection-site-specific indication while the EMA preferred a resistant-pathogen-specific indication for phase 3 trials of cefiderocol (31, 150). Such innovations in clinical trial design may enable the alignment of approved antibiotics with the unmet needs associated with antimicrobial resistance.

Structural, preclinical, and clinical data were inaccessible for several candidates. Considering that some public funding was critical for the early success of many candidates, we echo the call for broader data sharing (151). Although some public databases have compiled data, including ClinicalTrials.gov, the Pew Charitable Trusts, SPARK, and AntibioticDB, we should strive for completeness in archiving. As Achaogen, after declaring bankruptcy, shared its LpxC platform data with Forge Therapeutics, other abandoned data and learned lessons should be passed on.

In conclusion, the critical leak in the GNB-active antibiotic clinical development pipeline is between phase 1 and phase 2 and is largely attributable to safety issues. By sealing this rupture, we can increase the likelihood of FDA approval and de-risk investment in the antibiotic space. Given the complexities of antibiotic design from target validation and permeability to evasion of resistance mechanisms and nonconventional pharmacological properties, the low diversity of clinical trial termination reasons is notable. While safety presents a major challenge for antibiotic clinical development in this decade, solving this phase 1 issue may expose other issues in later clinical trials or after approval, like resistance or efficacy. Without innovations in preclinical predictive studies and clinical trial designs (148), the novel candidates in today’s preclinical pipeline that transition to clinical development in the next decade may face the same complications and consequences as those of the last. Alternatively, novel candidates with favorable *in vivo* profiles may be abandoned in the preclinical stage if the false-positivity rate of preclinical toxicity assays is too high. Finally, with many candidates withdrawn without public explanation as to why, it is challenging to learn from previous mistakes. Increased data sharing through existing mechanisms could reduce redundancy and accelerate future antibiotic development.
